# A flexible and efficient Bayesian implementation of point process models for spatial capture–recapture data

**DOI:** 10.1002/ecy.3887

**Published:** 2022-11-30

**Authors:** Wei Zhang, Joseph D. Chipperfield, Janine B. Illian, Pierre Dupont, Cyril Milleret, Perry de Valpine, Richard Bischof

**Affiliations:** ^1^ Department of Environmental Science, Policy and Management University of California Berkeley Berkeley California USA; ^2^ School of Mathematics and Statistics University of Glasgow Glasgow UK; ^3^ Faculty of Life Sciences and Natural Resource Management Norwegian University of Life Sciences Trondheim Norway; ^4^ Norwegian Institute for Nature Research Høyteknologisenteret Bergen Norway

**Keywords:** area search, binomial point process, continuous sampling, NIMBLE, non‐invasive genetic sampling, Poisson point process, spatial capture–recapture, wolverine

## Abstract

Spatial capture–recapture (SCR) is now routinely used for estimating abundance and density of wildlife populations. A standard SCR model includes sub‐models for the distribution of individual activity centers (ACs) and for individual detections conditional on the locations of these ACs. Both sub‐models can be expressed as point processes taking place in continuous space, but there is a lack of accessible and efficient tools to fit such models in a Bayesian paradigm. Here, we describe a set of custom functions and distributions to achieve this. Our work allows for more efficient model fitting with spatial covariates on population density, offers the option to fit SCR models using the semi‐complete data likelihood (SCDL) approach instead of data augmentation, and better reflects the spatially continuous detection process in SCR studies that use area searches. In addition, the SCDL approach is more efficient than data augmentation for simple SCR models while losing its advantages for more complicated models that account for spatial variation in either population density or detection. We present the model formulation, test it with simulations, quantify computational efficiency gains, and conclude with a real‐life example using non‐invasive genetic sampling data for an elusive large carnivore, the wolverine (*Gulo gulo*) in Norway.

## INTRODUCTION

Spatial capture–recapture (SCR) has found widespread application to the estimation of density and other sought‐after wildlife population parameters. SCR models (see Borchers & Fewster, 2016, for a review) extend traditional capture–recapture models by incorporating individual activity centers (ACs) into the modeling framework as latent variables. SCR models can, therefore, estimate spatially‐explicit abundance of a population. SCR models, like other hierarchical models in ecology, are increasingly implemented in a Bayesian framework due to the flexibility it affords, facilitated by accessible programming languages (de Valpine et al., 2017; Plummer, 2003). However, despite the growing sophistication and popularity of SCR, Bayesian practitioners are still faced with substantial computational challenges and a lack of effective tools to exploit existing features and new development, especially when dealing with large‐scale SCR problems.

Standard SCR models are composed of two hierarchical levels: one for modeling the number and distribution of ACs, and the other for modeling the number and distribution of detections of each individual conditional on the location of its AC and the location of detectors (e.g., traps or observers). Both the distribution of ACs and that of the detections can be modeled as spatial point processes (Efford, 2011). A spatial point process describes the distribution of points in space, with both the number and locations of points being random (Illian et al., 2008). Such models have been widely used for analyzing spatial data in diverse fields such as histology, epidemiology, and seismology among many others (Baddeley et al., 2006).

The distribution of ACs is already routinely modeled as a spatial point process since Efford (2004) described the first SCR model. A goal of many SCR models is to estimate and account for environmental factors that explain spatial variation in density, which can be achieved by fitting an inhomogenous point process. In Bayesian SCR, this is typically accomplished through the use of computationally inefficient categorical distributions and spatial density covariates, associated with a discrete habitat raster (Woodruff et al., 2021). Proffitt et al. (2015) described custom Markov chain Monte Carlo (MCMC) samplers for such models. The lack of tools for more efficient model fitting with inhomogeneous point processes in a Bayesian framework poses a computational bottleneck that can make large‐scale SCR analyses prohibitive (Milleret, Dupont, Brøseth, et al., 2018; Turek et al., 2021).

Furthermore, Bayesian SCR typically involves data augmentation (Royle et al., 2007), where completely unobserved individuals, their ACs, and their state (presence in the population) are imputed as part of the MCMC posterior sampling. This can be computationally costly, especially when detection rates are low and hence there may be many unobserved individuals. An alternative is to construct a semi‐complete data likelihood (SCDL), which does not require data augmentation (King et al., 2016). Although described and tested for simple SCR models by King et al. (2016), there are currently no tools readily available for implementing the SCDL approach in Bayesian SCR. Furthermore, it is yet unknown whether and to what extent the SCDL approach improves the computational efficiency of more complicated Bayesian SCR models. We address these issues in this paper.

The detection model in SCR depends on the type of detectors used for data collection. Most detection models to date were developed for sampling situations in which the set of possible detection locations is fixed (e.g., capture devices or camera traps); less common are detection models for area and transect searches (Royle, Kéry, & Guélat, 2011; Royle & Young, 2008). Although diverse, existing detector‐based and search‐encounter models do not adequately cover all common SCR sampling processes. For example, non‐invasive genetic sampling (NGS) data now commonly form the bases for SCR analyses (Bischof et al., 2020; López‐Bao et al., 2018). When NGS is implemented by searching a given area or along transects, paths taken by searchers can be recorded and used as a direct measure of effort in space and time. But due to technical and logistic limitations, or when samples are collected by the public, it is not always possible to know the spatial configuration of search effort. Detections are thus theoretically possible at any location within the general area that humans could visit. An analytical approach that uses actual detection locations would be preferable to the typical approach of projecting detections to an artificial detection grid (López‐Bao et al., 2018; Russell et al., 2012) as the latter: (1) means a potentially coarse approximation of detection locations, (2) involves aggregation of detection information, and (3) forces investigators to trade off precision for computational efficiency (Milleret, Dupont, Brøseth, et al., 2018).

Relying upon existing developments in SCR (e.g., Bischof et al., 2021; Efford, 2011; King et al., 2016), here we describe a hierarchical SCR model with point processes for both the ecological (AC distribution) and observational (detections) components. Specifically, we describe and provide tools for:

1. Efficient modeling of population density as an inhomogeneous point process. The model for AC distribution essentially represents second order habitat selection (placement of home ranges; Johnson (1980)) and can, in combination with other approaches for limiting computational burden, be applied to large‐scale SCR problems. We use simulations to evaluate the model's ability to reliably estimate density and coefficients associated with spatial covariates of density.
2. Modeling of detections in continuous space as an inhomogeneous point process. By eliminating the need for projecting detections to an artificial grid of detectors, this model represents more closely the spatially‐continuous data collection process. All spatial information in the data can be used without additional post‐collection approximation error. We use simulations to evaluate the model's ability to reliably estimate parameters, including coefficients associated with spatial covariates on detection probability. We also discuss the generality of the two approaches, in terms of ease of implementation and customization.
3. Performing SCR analysis without the need of data augmentation. We implement the SCDL approach for modeling density and expand upon the work in King et al. (2016) by evaluating it in the context of spatially varying density and detections across continuous space. We use simulations to test the ability of the model to produce reliable estimates of density. In addition, we compare the SCDL and data augmentation approaches for our point process SCR model in terms of computational efficiency.

Custom distributions and functions for implementing the aforementioned functionality are provided as part of a recently developed R package, *nimbleSCR* (Bischof et al., 2021). Aside from simulations to assess model performance, we demonstrate a real‐life application by fitting the model to NGS data of wolverines (*Gulo gulo*) and estimating density of this elusive large carnivore in a region of Norway.

## METHODS

### Population density as a point process

In SCR modeling, population density describes the number and spatial configuration of individual ACs. Spatial point processes have been widely used to model population density in SCR (Efford, 2004), with Poisson point processes being a common option (Borchers & Efford, 2008). In this case, population density is defined as the intensity function $\tilde{\lambda }\left(s|\beta \right)$ of the point process for the location s of ACs (indexed i below) with parameters β. Following Illian et al. (2008, p. 121), the log‐probability density of there being N ACs, $s_{1},\ldots ,s_{N}$, over the entire region $\tilde{o}$ of interest is

(1) $$\log\mathbb{P}\left(s_{1},\ldots ,s_{N}|\beta \right)\propto -\tilde{\Lambda }\left(\tilde{o}|\beta \right)+\sum_{i=1}^{N}\log\tilde{\lambda }\left(s_{i}|\beta \right),$$

where $\tilde{\Lambda }\left(\tilde{o}|\beta \right)=\int_{\tilde{o}}\tilde{\lambda }\left(s|\beta \right)ds$ is the expected number of ACs over $\tilde{o}$.

A homogeneous Poisson point process can be used, whose intensity function is constant across $\tilde{o}$. However, density of wildlife populations often varies in space as a result of various processes, including second‐order habitat selection, that is, home range placement (Johnson, 1980). With an inhomogeneous Poisson process, it is possible to model a spatially varying intensity surface and thus population density as a function of spatial covariates. To achieve this, $\tilde{o}$ is divided into a set of H non‐overlapping windows $\left\{{\tilde{w}}_{1},\ldots ,{\tilde{w}}_{H}\right\}$, within each of which the value of a covariate is constant. Covariates are then related to the intensity value of each window through some link function. In principal, any non‐negative function is valid for this purpose. Here, we consider the log‐linear model to define ${\tilde{\lambda }}_{h}$, the intensity value of window ${\tilde{w}}_{h},h=1,\ldots ,H$: $\log({\tilde{\lambda }}_{h})=\beta_{0}+\sum_{i}\beta_{i}\xi_{\mathrm{ih}}\;$ where $\xi_{\mathrm{ih}}$ is the value of the i‐th covariate in window ${\tilde{w}}_{h}$. The spatial resolution and extent of the windows can be easily chosen to match the scale at which habitat selection occurs, and allow the fitting of any additive or interactive effects between spatial covariates.

A known result of the Poisson point process is that conditioning on the total number of points in a given region yields a binomial point process (p. 69, Illian et al., 2008), a point process with a fixed number of points. For a binomial point process, the probability density of one point's location is the intensity of the corresponding Poisson process evaluated at that point divided by the integral of the intensity function over the entire region. It follows that, given individual i exists in the population, the probability density that it has AC $s_{i}$ is the probability density of a binomial point process with a single point, i.e., a Bernoulli point process:

(2) $$\mathbb{P}\left(s_{i}|\beta \right)=\frac{\tilde{\lambda }\left(s_{i}|\beta \right)}{\tilde{\Lambda }\left(\tilde{o}|\beta \right)}.$$

Thus, the log‐probability density for the AC $s_{i}$ of detected individual i is

(3) $$\log\mathbb{P}\left(s_{i}|\beta \right)=-\log\tilde{\Lambda }\left(\tilde{o}|\beta \right)+\log\tilde{\lambda }\left(s_{i}|\beta \right).$$

Using Equation (3) to model the location of individual ACs offers an alternative to the computationally inefficient categorical distribution typically used in Bayesian SCR models with inhomogeneous density. In that approach, each $s_{i}$ is placed in a grid cell and then either a uniform distribution is used for the exact location within the grid cell or the centroid of the cell is assigned as an approximate location. The latter is more efficient for model fitting and is often adopted when there are a relatively large number of grid cells so that the approximation works well. In either of the two schemes, using the categorical distribution results in inefficient MCMC steps, especially when the number of cells is large. This is because the categorical MCMC sampler implemented in commonly used software packages (e.g., *nimble* and JAGS) iterates over each of the grid cells when sampling the cell index for each individual in each MCMC iteration. However, when the Bernoulli point process is used for AC distribution, calculations of intensity values for all cells need to be done only once in each MCMC iteration. In *nimble* sampling the AC location of each individual uses a block random walk sampler, that is, an adaptive Metropolis‐Hastings algorithm with a multivariate normal proposal distribution. Our approach does not require any approximation in the process of model fitting. In addition, our approach eliminates the need of the so‐called ones or zeros trick that is typically used when modeling density across an irregular (i.e., non‐rectangular or non‐contiguous) habitat in Bayesian SCR models.

### Detection as a point process

When SCR data are collected by searching over a region o, the location of each detection can be any point y in o. Similar to population density, the distribution of these detection locations can be described as a point process with intensity $\lambda \left(y|s,\theta ,\sigma \right)$, which can be split into two parts: $\lambda \left(y|s,\theta ,\sigma \right)=b\left(y|\theta \right)\;\tau \left(y|s,\sigma \right),$ where $\tau \left(y|s,\sigma \right)$ is a kernel with parameters σ, describing the distance decay relationship of the detection intensity from the AC s, and $b\left(y|\theta \right)$ denotes the baseline detection intensity with parameters θ, which may or may not vary across the detection region o. The kernel $\tau \left(y|s,\sigma \right)$ can be considered as a functional description of the home range of the individual with AC s. Heterogeneity in detection probability might result from differences in: (1) landscape characteristics affecting detectability, (2) sampling effort, and (3) detector efficiency (Efford et al., 2013). Similar to the point process for density, it is possible to model this spatially varying intensity of the detection point process by using spatial covariates. We can divide the detection region o into a set of L non‐overlapping detection windows $w_{1},\ldots ,w_{L}$, within each of which the value of each covariate is constant. Again, we consider the log‐linear model and define $b_{l}$ to be the baseline detection intensity of window $w_{l},l=1,\ldots ,L$: $\log\left(b_{l}\right)=\theta_{0}+\sum_{i}\theta_{i}\zeta_{\mathrm{il}},$ where $\zeta_{\mathrm{il}}$ is the value of the i‐th detection covariate within window $w_{l}$.

Following Equation (1), the log‐probability density for the $M_{i}$ detection locations, $y_{i,1}\ldots y_{i,M_{i}}$, of individual i with AC $s_{i}$ is:

(4) $$\log\mathbb{P}\left(y_{i,1},\ldots ,y_{i,M_{i}}|s_{i},\theta ,\sigma \right)=-\Lambda \left(o|s_{i},\theta ,\sigma \right)+\sum_{j=1}^{M_{i}}\log\lambda \left(y_{i,j}|s_{i},\theta ,\sigma \right),$$

where $\Lambda \left(o|s_{i},\theta ,\sigma \right)=\int_{o}\lambda \left(y|s_{i},\theta ,\sigma \right)dy$ is the expected number of detections of individual i over the entire detection region o. Assuming o is composed of a single polygon, our sampling situation is identical to that described in Efford (2011). However, modeling detections explicitly as a spatial point process integrates detection and density sub‐models of SCR into a unified framework, similar to Yuan et al. (2017) where an integrated approach used point process models for distance sampling data.

Under the detection point process presented above, we provide several options to accommodate different SCR sampling scenarios in practice. The first option is using Equation (4) to fit data that typically arise from area searches for DNA samples of animals, where any individual can be detected more than once within the detection region. The second option is modeling the case where only a single detection per individual is possible within o (e.g., dead recoveries, Dupont et al., 2021): $\log\mathbb{P}\left(y_{i}|s_{i},\theta ,\sigma \right)=-\log\Lambda \left(o|s_{i},\theta ,\sigma \right)+\log\lambda \left(y_{i}|s_{i},\theta ,\sigma \right).$ If this kind of sampling is repeated over a number of occasions, individual detection locations can be modeled using a binomial point process.

### SCDL approach

It is popular to fit Bayesian SCR models via data augmentation to estimate the abundance and population density including never‐observed individuals (Royle et al., 2007). An alternative is to use the SCDL approach of King et al. (2016), where AC locations of undetected individuals are integrated out from the complete data likelihood (Little & Rubin, 1983) so that there is no need to use data augmentation to fit the model. Using this approach, King et al. (2016) investigated a set of capture–recapture models with individual heterogeneity, including a simple SCR model. Here, we expand the approach to more complicated cases and provide functionalities to fit Bayesian SCR models without data augmentation.

Key to the SCDL approach is to calculate the marginal void probability $p^{*}$ that individual i is never detected. This calculation requires the probability density function $\mathbb{P}\left(s_{i}|\beta \right)$ of the individual's AC $s_{i}$ given in Equation (2) and the probability $\mathbb{P}\left(D_{i,o}=0|s_{i},\theta ,\sigma \right)$ that the individual is never detected conditional on its AC, where $D_{i,o}$ denotes the number of detections of individual i over the region o. Note that $\mathbb{P}\left(D_{i,o}=0|s_{i},\theta ,\sigma \right)=\exp\left\{-\Lambda \left(o|s_{i},\theta ,\sigma \right)\right\}$ can be obtained by setting $M_{i}$ to be 0 in Equation (4) and back‐transforming the equation. Then it follows that $p^{*}$ can be expressed as

(5) $$\begin{array}{ll} p^{*}=\mathbb{P}\left(D_{i,o}=0|,\theta ,,,\sigma ,,,\beta \right) & =\int_{\tilde{o}}\mathbb{P}\left(s_{i},|,\beta \right)\mathbb{P}\left(D_{i,o}=0|s_{i},\theta ,\sigma \right)ds_{i}\\ & =\frac{1}{\tilde{\Lambda }\left(\tilde{o},|,\beta \right)}\int_{\tilde{o}}\tilde{\lambda }\left(s_{i}|\beta \right)\exp\left\{-\Lambda \left(o|s_{i},\theta ,\sigma \right)\right\}ds_{i}. \end{array}$$

Since individual heterogeneity is not considered here, $p^{*}$ is the same for all individuals in the population; otherwise $p^{*}$ needs to be calculated for each individual in each MCMC iteration.

Calculating $p^{*}$ seems complicated and involves calculations of three definite integrals; however, it can often be simplified in practice. In Appendix S1: Section S1, we describe how the intensity of the Poisson process for AC distribution can be written as a piece‐wise constant function. We then introduce in Appendix S1: Section S2 the commonly used Gaussian kernel function for the detection process and describe how the baseline detection intensity can be written as a piece‐wise constant function. It follows that $p^{*}$ in Equation (5) is simplified and only involves calculating one definite integral, which is obtained numerically using the midpoint rule; see Appendix S1: Section S3 for more details. Nevertheless, the computational cost of the $p^{*}$ makes it unclear whether the SCDL approach will be more efficient than data augmentation in all cases.

### Software implementation

We provide R functions for the aforementioned models in package *nimbleSCR* (Bischof et al., 2021; Turek et al., 2021). These functions allow one to flexibly formulate Bayesian hierarchical point process SCR models tailored to their situation. In addition, to fit the model one can choose between the SCDL approach and data augmentation.

Assembled models can be fitted to data using Bayesian MCMC in R package *nimble* (de Valpine et al., 2017). *nimble* supports nearly the same modeling language as JAGS and WinBUGS but allows extensions with new functions and distributions. We used these capabilities to write both Poisson and binomial point process distributions and also marginal void probability calculations in *nimble*, allowing them to be efficiently and automatically incorporated into MCMC sampling. Where feasible, *nimble* functions provided here already include features that have proven to substantially boost computational efficiency of Bayesian SCR models (Milleret, Dupont, Bonenfant, et al., 2018; Turek et al., 2021), allowing fitting of complex models and application to large‐scale estimation problems (Bischof et al., 2020).

## RESULTS

### Model validation

We first conducted a simulation study to investigate the relative bias and frequentist coverage of credible intervals for calibrated Bayes interpretation (Little, 2006) under a range of covariate effects. We set the habitat region $\tilde{o}$ to be a 10 × 10 km square, which included a buffer of 0.6 km around the 8.8 × 8.8 km square detection region o. We divided $\tilde{o}$ into 100 equally‐sized windows and o into 25 equally‐sized windows. We considered two spatial covariates, one for modeling the intensity for population density and the other for the baseline detection intensity. Values of both covariates were sampled from a uniform distribution: $\;\text{Uniform}\;\left(-1,1\right)$. The intercept parameters $\beta_{0}$ and $\theta_{0}$ were set to be 1 and 2, respectively. The values of the slope parameters $\beta_{1}$ and $\theta_{1}$ were chosen from set $\left\{-1,0,1\right\}$ so that there were nine scenarios defined by the different combinations of the two parameters. An isotropic multivariate Gaussian function with σ=0.2 was used as the detection kernel. For each scenario, we simulated 100 datasets under the hierarchical point process model. The model was then fit to the data using Bayesian MCMC in *nimble*. MCMC sampling was run for 110,000 iterations with the first 10,000 discarded as burn‐in. The population size estimator was unbiased with roughly nominal values for the credible interval coverage in all scenarios; see Figure S1 in Appendix S3. Similar conclusions can be drawn from the estimation results of other parameters. We, therefore, conclude that the proposed model works well for parameter estimation.

### Benefits of modeling population density as point process

We performed another simulation study to compare the efficiency of the point process model for population density and the categorical distribution typically used in Bayesian SCR models. We assumed the population size to be N=100 in the simulation. The habitat region $\tilde{o}$ was a 12 × 12 km square, including a buffer of 2 km around the 8 × 8 km detection region o divided in 64 equally sized detection windows. The habitat region $\tilde{o}$ was divided into 144, 36, 9, and 4 equally‐sized windows in four simulation scenarios. One spatial covariate was used to model the intensity of population density. ACs were simulated using the Bernoulli point process. We used the Poisson point process model to generate detection data. Data augmentation was used for model fitting, and thus we did not estimate parameter $\beta_{0}$. The slope parameter $\beta_{1}$ was set to 2. We set slope parameter $\theta_{1}$, and the intercept parameter $\theta_{0}$ to be −1. An isotropic multivariate Gaussian function with σ=1 was used as the detection kernel.

**FIGURE 1:**
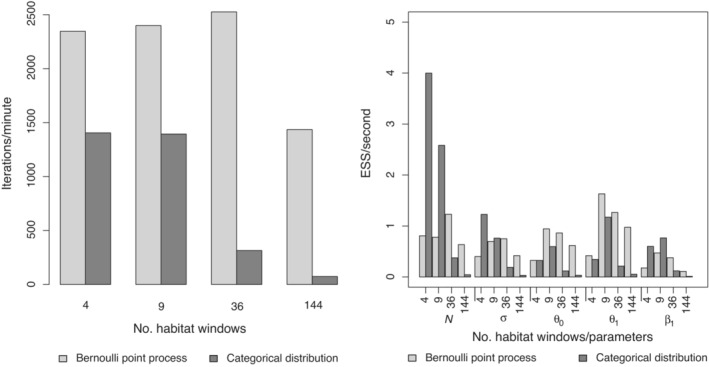
Results from simulations comparing the Bernoulli point process model and the categorical distribution used for activity center (AC) distribution in Bayesian spatial capture–recapture (SCR) models. Two models are compared in terms of the number of MCMC iterations per minute (left panel) and effective sample size per second for each model parameter (right panel).

The simulation results are summarized in Figure 1. We measured the efficiency of MCMC via the effective sample size per second. We found that increasing the habitat resolution (more windows) drastically decreased the efficiency of MCMC and exponentially increased the runtime of the model using the categorical distribution, while runtime remained comparatively low regardless of the number of habitat windows when using the point process model for density.

### SCDL versus data augmentation

We performed a third simulation study to compare the efficiency of the SCDL approach and the more common data augmentation (DA) approach for the hierarchical point process SCR model. We set the habitat region $\tilde{o}$ to be a 12 × 12 km square, including a buffer of 2 km around a 8 × 8 km square detection region o. As we expected the computation time of $p^{*}$, and thus the efficiency of the SCDL approach, to be strongly affected by the number of both habitat and detection windows, we considered different resolutions for $\tilde{o}$ (1, 2, 4, and 6 km) and o (1 and 4 km), leading to scenarios with 144, 36, 9, or 4 habitat windows, and 64 or 4 detection windows. The intensities for population density and the detection process were modeled as functions of spatial covariates. Covariate values for each of the habitat and detection windows were sampled from a uniform distribution: $\;\text{Uniform}\;\left(-1,1\right)$. The intercept parameters $\beta_{0}$ and $\theta_{0}$ were set to be 1 and −1, respectively. The slope parameters $\beta_{1}$ and $\theta_{1}$ were set to −1 and 2, respectively. An isotropic multivariate Gaussian function with σ=1 was used as the detection kernel.

For each of the eight scenarios, we simulated 100 individual ACs using the Bernoulli point process in Equation (3). Detections were simulated using the detection point process in Equation (4). For the SCDL approach, we varied the number of nodes used in the calculation of $p^{*}$ (4, 25, and 100; see Appendix S1: Section S3) to investigate the effect of the numerical integration on overall efficiency. For the DA approach, we varied the augmentation factor (2, 3, and 5). MCMC sampling was run for 6000 iterations with the first 1000 discarded as burn‐in.

**FIGURE 2:**
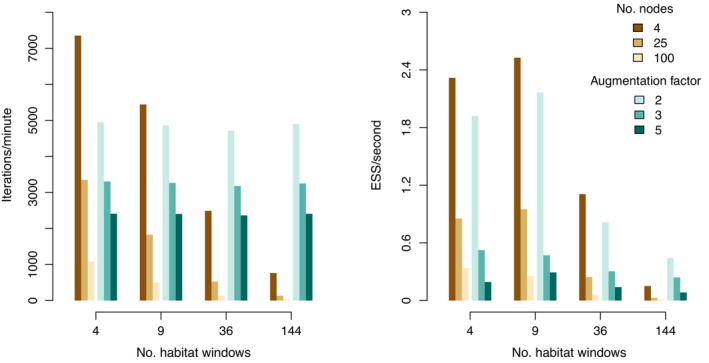
Simulation results comparing the semi‐complete data likelihood (SCDL) approach (brown colors) and the data augmentation (DA) approach (turquoise colors) for fitting the point process spatial capture–recapture (SCR) model. Two approaches are compared in terms of the number of MCMC iterations per minute (left panel) and minimum effective sample size per second of all model parameters (right panel).

We found that SCDL is more efficient than DA when the habitat and detection regions consist of a small number of windows (≤ 36 in our setup); see Figure 2. As the number of windows increases, DA becomes more efficient than SCDL. In addition, the number of nodes used to calculate $p^{*}$ via numerical integration has a strong negative impact on the efficiency of the SCDL approach. This is problematic as a greater number of nodes used in numerical integration means a more accurate approximation of $p^{*}$. By contrast, the efficiency of DA appears to be less sensitive to the augmentation factor. We conclude that SCDL can be a powerful approach to fit simple SCR models, where dividing either the habitat or detection region is not necessary (i.e., absence of spatial variation in either density or detectability). However, SCDL seems to lose its advantages when the model becomes complicated and spatial variation exists in the process of either population density or detection.

### Wolverine NGS data analysis

Finally, to demonstrate the application of the proposed model to real data problems, we applied it to part of the wolverine data available in the Scandinavian large carnivore database, Rovbase 3.0 (http://rovbase.se/ or http://rovbase.no/). This database is jointly used by Norway and Sweden to record NGS data, dead recoveries, GPS search tracks, and observations of wolverines and other large carnivores. We analyzed a subset of the Rovbase dataset composed of female wolverines detected from December 2018 to June 2019 in Norwegian counties of Hedmark, Oppland, and parts of Sør‐Trøndelag. The dataset is composed of 228 scat‐ and hair‐based DNA samples from 72 female wolverines.

The detection region o was divided into L=195 windows each of size 20 × 20 km and the habitat region $\tilde{o}$ was divided into H=40 windows each of size 60 × 60 km. The region $\tilde{o}$ covers the entire region o and a surrounding buffer, allowing the ACs of individuals to be located outside the searched area. The number of known wolverine dens was used as a covariate for modeling population density (Bischof et al., 2020). Four covariates were used for modeling baseline detection: the detection location, the recorded length of GPS tracks logged by searchers, the average percentage of snow cover (MODIS at 0.1 degrees resolution, www.neo.sci.gsfc.nasa.gov, accessed 10 November 2019), and the average distance to the nearest primary and secondary roads.

To investigate how the point process method presented in this paper compares with commonly used methods, we analyzed the data in four different ways as shown in Table 1. For population density, we considered either the Bernoulli point process or the categorical distribution where the center of each habitat grid cell is regarded as the approximate AC location when an individual is located in that cell. For detection, we considered either the Poisson process or the commonly used discrete detector approach where each detection grid cell is regarded as a “detector” (Milleret, Dupont, Brøseth, et al., 2018). For model fitting, we considered either the SCDL approach or the DA approach. In each of the four scenarios in Table 1, we ran four MCMC chains for 11,000 iterations and removed the first 1000 iterations as burn‐in. MCMC convergence was checked and confirmed using R‐hat values and visual inspections of trace plots.

**TABLE 1:** Four different ways to analyze the wolverine data and their estimation results (posterior means and 95% credible intervals) of key parameters N and σ.

Method	AC distribution	Detection	Fitting	N	ESS^N^/s	σ	ESS^σ^/s
1	Bernoulli	Poisson process	SCDL	137 (115,163)	0.23	5.22 (4.81, 5.66)	0.07
2	Categorical	Discrete detector	DA	109 (94,127)	0.98	20.73 (19.14, 22.52)	0.48
3	Bernoulli	Poisson process	DA	139 (116,166)	0.51	5.23 (4.81, 5.69)	0.37
4	Categorical	Poisson process	DA	98 (86,113)	0.27	19.77 (18.35, 21.36)	0.15

*Note*: ESS/s denotes effective sample size per second.

Abbreviations: AC, activity center; DA, data augmentation; ESS, effective sample size; SCDL, semi‐complete data likelihood.

In Table 1, we present the estimation results of two key parameters of interest, abundance N and the detection kernel parameter σ regulating home range size. Both methods using the unified point process framework produced similar results for both parameters. However, the point‐process DA approach cost 1.2 h, in contrast to 6.8 h required by the point‐process SCDL approach. We assessed the efficiency of the two fitting approaches via the effective sample size per second (ESS/s) for the two parameters. The DA approach (ESS/s 0.51 and 0.37 for N and σ) is more efficient than the SCDL approach (ESS/s 0.23 and 0.07 for N and σ), which is consistent with what was observed in the simulation studies. Comparing the third and fourth methods in the table, we can see that the Bernoulli point process makes model fitting more efficient than the categorical distribution. This is also consistent with our observation in the simulation studies. Compared to the unified point process methods, the other two methods yielded higher estimated values of σ and lower values of N. This is not surprising, given that AC locations are approximated by grid cell centers when the categorical distribution is used for AC distributions and the continuous detection process is approximated using the discrete detector approach (Milleret, Dupont, Brøseth, et al., 2018). Using the two approximations together (the second method) yielded the best computational efficiency; however, this was achieved at the cost of losing accuracy for parameter estimation. The estimation results could be closer to those obtained by the unified point process methods if the resolutions of the habitat and detection grids are set higher. However, this means a larger number of grid cells and lower computational efficiency. Estimation results of other model parameters can be found in Appendix S2.

## DISCUSSION

Point processes are useful representations of the main processes from which SCR data arise (Efford, 2004, 2011). The custom distributions and functions described here have been added to R package *nimbleSCR* (Bischof et al., 2021; Turek et al., 2021), allowing practitioners to easily build and fit point process SCR models in *nimble* (de Valpine et al., 2017). Furthermore, spatial covariates can be readily incorporated to model the intensity functions of the inhomogeneous point processes for both population density and detection. Aside from making these modeling tools accessible for a wider range of applications, the implementation in *nimble* also offers functionality for improved computational efficiency (Milleret, Dupont, Bonenfant, et al., 2018), one of the main obstacles to the analysis of large‐scale monitoring data with SCR (Bischof et al., 2020). Practitioners can assemble SCR models using all or a subset of the distributions and functions we described in this paper.

We found that the Bernoulli point process for AC distribution outperforms the conventionally used categorical distribution in Bayesian SCR models, especially with a larger number of habitat windows. This was one of the factors that enabled us to fit SCR models to data from the entire Scandinavian range of three large carnivore species (Bischof et al., 2020). The Poisson point process for detection is a suitable choice when detections, at least in theory, are possible anywhere within a defined region (such as opportunistic NGS). Even in cases where the detection process itself is better modeled using discrete detectors or a search‐encounter model (Royle, Magoun, et al., 2011), the flexibility of the point process framework facilitates the expansion of closed‐population SCR to open‐population SCR (OPSCR) models. Incorporating AC movement between occasions and dead recovery data into OPSCR analysis can be easily achieved using the Bernoulli point process. We have added functions for this into the *nimbleSCR* package.

The SCDL approach allows one to fit Bayesian SCR models without the need for data augmentation. However, computational inefficiency, arising from the numerical integration needed for computing the void probability, makes this approach impractical for applications that include a moderate number of detection windows. Furthermore, in its current form, SCDL is not suitable for OPSCR models, whereas data augmentation is readily incorporated.

## CONFLICT OF INTEREST

The authors declare no conflict of interest.

## Supporting information


Appendix S1

Appendix S2

Appendix S3

## Data Availability

Novel statistical code and the wolverine data (Zhang et al., 2022) used in the paper are available in Zenodo at https://doi.org/10.5281/zenodo.7038425.
