# Supplementary material for: A flexible and efficient Bayesian implementation of point process models for spatial capture–recapture data
Source: Ecology. 2022 Nov 30;104(1):e3887. doi: 10.1002/ecy.3887 (PMC10078592; doi:10.1002/ecy.3887)
Supplement: Supplementary file 1 — Appendix S1 [file ECY-104-0-s003.pdf]

Appendix S1: A flexible and efficient Bayesian implementation of point process models for spatial capture-recapture data

Zhang W., J.D. Chipperfield, J.B. Illian, P. Dupont, C. Milleret,
P. de Valpine, R. Bischof

Ecology

Section S1 Cell-based AC distribution

Note that $\tilde{\lambda}_h$ is the intensity value for population density of window $\tilde{\boldsymbol{w}}_h, h = 1, \ldots, H$. It follows that the population density intensity function $\tilde{\lambda}(\boldsymbol{s}|\boldsymbol{\beta})$ can be written as

$$\tilde{\lambda}(\boldsymbol{s}|\boldsymbol{\beta}) = \sum_{h=1}^{H} \tilde{\lambda}_h \mathscr{I}(\boldsymbol{s} \in \tilde{\boldsymbol{w}}_h), \quad \text{(S1)}$$

where $\mathscr{I}(\cdot)$ is the usual indicator function that equals 1 if the condition $\cdot$ is true and 0 otherwise.

Provided that an inhomogeneous Poisson point process is used for population density, the number of ACs $N_{\tilde{\boldsymbol{w}}}$ within a subregion $\tilde{\boldsymbol{w}} \subset \tilde{\boldsymbol{o}}$ follows a Poisson distribution:

$$N_{\tilde{\boldsymbol{w}}} \sim \text{Poisson}\left\{\tilde{\Lambda}(\tilde{\boldsymbol{w}}|\boldsymbol{\beta})\right\}, \quad \text{(S2)}$$

where

$$\tilde{\Lambda}(\tilde{\boldsymbol{w}}|\boldsymbol{\beta}) = \int_{\tilde{\boldsymbol{w}}} \tilde{\lambda}(\boldsymbol{s}|\boldsymbol{\beta}) d\boldsymbol{s} \quad \text{(S3)}$$

is the expected number of ACs located within $\tilde{\boldsymbol{w}}$. According to Eq. S3, the expected number of ACs within window $\tilde{\boldsymbol{w}}_h$ is

$$\tilde{\Lambda}(\tilde{\boldsymbol{w}}_h|\boldsymbol{\beta}) = \tilde{\lambda}_h V_h, \quad \text{(S4)}$$

where V_h is the area of window $\tilde{\boldsymbol{w}}_h$. It follows that the expected total number of ACs across the entire

region $\tilde{\boldsymbol{o}}$ is

$$\tilde{\Lambda}(\tilde{\boldsymbol{o}}|\boldsymbol{\beta}) = \sum_{h=1}^{H} \tilde{\Lambda}(\tilde{\boldsymbol{w}}_h|\boldsymbol{\beta}) = \sum_{h=1}^{H} \tilde{\lambda}_h V_h. \tag{S5}$$

Section S2 Cell-based detection

Note that b_l denotes the baseline detection intensity of window $\boldsymbol{w}_l, l = 1, \ldots, L$. Then, similarly to the population density case above, the baseline detection intensity function can be written as

$$b(\boldsymbol{y}|\boldsymbol{\theta}) = \sum_{l=1}^{L} b_l \mathscr{I}(\boldsymbol{y} \in \boldsymbol{w}_l). \tag{S6}$$

A common detection decay kernel is the isotropic multivariate Gaussian kernel

$$\begin{aligned} \tau(\boldsymbol{y}|\boldsymbol{s}_i, \sigma) &= \exp\left\{-\frac{1}{2\sigma^2}\sum_{j=1}^{2}\left(y_j - s_{ij}\right)^2\right\} \\ &= (2\pi)\prod_{j=1}^{2}\phi\left(\frac{y_j - s_{ij}}{\sigma}\right), \end{aligned} \tag{S7}$$

where $\phi(x)$ is the standard normal probability density function

$$\phi(x) = \frac{1}{\sqrt{2\pi}}\exp\left(-\frac{x^2}{2}\right). \tag{S8}$$

Here, $\tau(\boldsymbol{y}|\boldsymbol{s}_i, \sigma)$ is proportional to the probability density function of a multivariate Gaussian distribution with mean equal to the activity centre $\boldsymbol{s}_i$ of individual i, and with a diagonal variance-covariance matrix whose diagonal elements are all set to σ^2, representing the isotropic variance of the detection decay kernel. Thus, the parameter σ regulates the home range size of the species of interest. In this paper, we do not consider covariates for σ. However, it is immediate that σ can be modeled as a regression of relevant environmental and/or individual covariates allowing for different home range sizes in different environments or for species that have different home ranges at different life stages or between different sexes.

Inserting the cell-based baseline detection function Eq. S6 and the Gaussian decay kernel Eq. S7 into Eq. 5 in the main text yields the following composite detection intensity function

$$\lambda\left(\boldsymbol{y}|\boldsymbol{s}_i,\boldsymbol{\theta},\sigma\right) = (2\pi)\left\{\sum_{l=1}^{L} b_l \mathscr{I}\left(\boldsymbol{y}\in\boldsymbol{w}_l\right)\right\}\prod_{j=1}^{2}\phi\left(\frac{y_j - s_{ij}}{\sigma}\right). \tag{S9}$$

Section S3 Marginal void probability

Similar to the point process for AC distribution, the number of detections $D_{i,\boldsymbol{w}}$ of individual i within a subregion $\boldsymbol{w}\subset\boldsymbol{o}$ follows a Poisson distribution:

$$D_{i,\boldsymbol{w}} \sim \text{Poisson}\left\{\Lambda\left(\boldsymbol{w}|\boldsymbol{s}_i,\boldsymbol{\theta},\boldsymbol{\sigma}\right)\right\}, \tag{S10}$$

where

$$\Lambda\left(\boldsymbol{w}|\boldsymbol{s}_i,\boldsymbol{\theta},\boldsymbol{\sigma}\right) = \int_{\boldsymbol{w}}\lambda\left(\boldsymbol{y}|\boldsymbol{s}_i,\boldsymbol{\theta},\boldsymbol{\sigma}\right)d\boldsymbol{y} \tag{S11}$$

is the expected number of detections within $\boldsymbol{w}$. Then the expected number of detections $\Lambda\left(\boldsymbol{w}_l|\boldsymbol{s}_i,\boldsymbol{\theta},\sigma\right)$ of individual i within window $\boldsymbol{w}_l$ is

$$\begin{aligned}\Lambda\left(\boldsymbol{w}_l|\boldsymbol{s}_i,\boldsymbol{\theta},\sigma\right) &= (2\pi)\, b_l\prod_{j=1}^{2}\int_{w_{lj1}}^{w_{lj2}}\phi\left(\frac{y_j - s_{ij}}{\sigma}\right)dy_j \\ &= (2\pi)\,\sigma^2 b_l\prod_{j=1}^{2}\left[\Phi\left(\frac{w_{lj2}-s_{ij}}{\sigma}\right)-\Phi\left(\frac{w_{lj1}-s_{ij}}{\sigma}\right)\right],\end{aligned} \tag{S12}$$

where w_{lj1} and w_{lj2} are the lower and upper bounds of the j-th dimension of window $\boldsymbol{w}_l$, and $\Phi(\cdot)$ is the standard Gaussian cumulative distribution function. Then the expected number of detections of individual i across the entire region $\boldsymbol{o}$ is

$$\Lambda\left(\boldsymbol{o}|\boldsymbol{s}_i,\boldsymbol{\theta},\sigma\right) = (2\pi)\,\sigma^2\sum_{l=1}^{L} b_l\prod_{j=1}^{2}\left[\Phi\left(\frac{w_{lj2}-s_{ij}}{\sigma}\right)-\Phi\left(\frac{w_{lj1}-s_{ij}}{\sigma}\right)\right]. \tag{S13}$$

According to Eq. 8 in the main text, the marginal void probability in the case of cell-based AC distribution and detection is

$$\begin{aligned}\mathbb{P}\left(D_{i,\boldsymbol{o}}=0|\boldsymbol{\beta},\boldsymbol{\theta},\sigma,i\leq N\right)&=\frac{1}{\tilde{\Lambda}\left(\tilde{\boldsymbol{o}}|\boldsymbol{\beta}\right)}\int_{\tilde{\boldsymbol{o}}}\tilde{\lambda}\left(\boldsymbol{s}_i|\boldsymbol{\beta}\right)\exp\left\{-\Lambda\left(\boldsymbol{o}|\boldsymbol{s}_i,\boldsymbol{\theta},\sigma\right)\right\}d\boldsymbol{s}_i\\&=\frac{1}{\tilde{\Lambda}\left(\tilde{\boldsymbol{o}}|\boldsymbol{\beta}\right)}\sum_{h=1}^{H}\tilde{\lambda}_h\int_{\tilde{\boldsymbol{w}}_h}\exp\left\{-\Lambda\left(\boldsymbol{o}|\boldsymbol{s}_i,\boldsymbol{\theta},\sigma\right)\right\}d\boldsymbol{s}_i,\end{aligned} \tag{S14}$$

where $\tilde{\Lambda}\left(\tilde{\boldsymbol{o}}|\boldsymbol{\beta}\right)$ is given in Eq. S5, and the definite integral $\int_{\tilde{\boldsymbol{w}}_h}\exp\left\{-\Lambda\left(\boldsymbol{o}|\boldsymbol{s}_i,\boldsymbol{\theta},\sigma\right)\right\}d\boldsymbol{s}_i$ can be calculated numerically using the midpoint rule.

To obtain the marginal void probability Eq. S14, we compute the integral

$$I_h=\int_{\tilde{\boldsymbol{w}}_h}\exp\left\{-\Lambda\left(\boldsymbol{o}|\boldsymbol{s}_i,\boldsymbol{\theta},\sigma\right)\right\}d\boldsymbol{s}_i \tag{S15}$$

numerically using the 2-dimensional midpoint rule. For convenience, we denote the integrand by

$$f(\boldsymbol{s}_i)=\exp\left\{-\Lambda\left(\boldsymbol{o}|\boldsymbol{s}_i,\boldsymbol{\theta},\sigma\right)\right\} \tag{S16}$$

and aim to integrate this function over each of the H habitat windows.

The 2-dimensional midpoint rule starts by dividing the habitat window $\tilde{\boldsymbol{w}}_h$ into a set of rectangular subregions. We let these subregions be of the same size, which, however, is not required by the method itself. Both the x- and y-dimensions of the window are divided into m equal subintervals. This results in a set of $n=m^2$ rectangular subregions of equal size. The center point of each rectangle is taken as a node for numerical integration. This leads to a set of n nodes $\{\boldsymbol{\epsilon}_1,\ldots,\boldsymbol{\epsilon}_n\}$. Then the integral can be computed by

$$\tilde{I}_h=\frac{s_h}{n}\sum_{i=1}^{n}f(\boldsymbol{\epsilon}_i), \tag{S17}$$

where s_h is the area of habitat window $\tilde{\boldsymbol{w}}_h$.
