# Supplementary material for: A flexible and efficient Bayesian implementation of point process models for spatial capture–recapture data
Source: Ecology. 2022 Nov 30;104(1):e3887. doi: 10.1002/ecy.3887 (PMC10078592; doi:10.1002/ecy.3887)
Supplement: Supplementary file 2 — Appendix S2 [file ECY-104-0-s001.pdf]

Appendix S2: A flexible and efficient Bayesian implementation of point process models for spatial capture-recapture data

Zhang W., J.D. Chipperfield, J.B. Illian, P. Dupont, C. Milleret, P. de Valpine, R. Bischof

Ecology

Section S1 Wolverine data

R. Bischof was given specific access privileges to Rovbase by the Norwegian Environment Agency to be able to download the data for the analysis shown in this paper. The download date was 2019-10-08. We provide all the formatted data used in the analysis on GitHub so that others can repeat the analysis.

The downloaded data consist of two parts: 1) all dead recovery data for wolverine (if identified as such based on genetic analysis) entered into the database since the start of collection until the download date and 2) all genetic sampling data for wolverine (if identified as such based on genetic analysis) entered into the database since the start of collection until the download date. We used the genetic sampling data for the analysis. The primary fields we used were individual ID (from genotyping), sex, sample or carcass coordinates, collection date, and entity that submitted the sample. The data and analysis code (Zhang et al., 2022) can be found in Zenodo at https://doi.org/10.5281/zenodo.7038425.

Section S2 Further results of the wolverine data analysis

Here we provide further results of the wolverine data analysis described in the paper. Fig. S1 presents the distribution of the non-invasive DNA samples of female wolverines collected during the winter of 2018/19 in Norway, violin plots of the posterior samples of abundance N obtained from the four different methods used to analyze the wolverine data, and the effective sample sizes per second for N using the four methods.

Tables S1–S4 present the estimation results of model parameters for each of the four different

methods applied to analyze the wolverine data. The results of parameters N and σ are shown in the paper and thus omitted here. In the tables, θ_1, θ_2, and θ_3 denote respectively the intercepts for detection locations Hedmark, Oppland, and parts of Sør-Trøndelag. θ_4, θ_5, and θ_6 denote respectively the coefficients of three detection covariates: the recorded length of GPS tracks logged by searchers, the average percentage of snow cover, and the average distance to the nearest primary and secondary roads. β_0 and β_1 denote the intercept and slope of the log-linear model for modeling population density using the number of known wolverine dens as a single covariate. Note that when fitting the model using the data augmentation approach, β_0 is not included in the model and thus its results are not given in Tables S2–S4. ψ is a parameter defined for model fitting using data augmentation, and denotes the probability that an individual in the augmented super-population is actually a member of the population exposed to sampling. The super-population size M was set to be 300 for fitting the models using data augmentation.

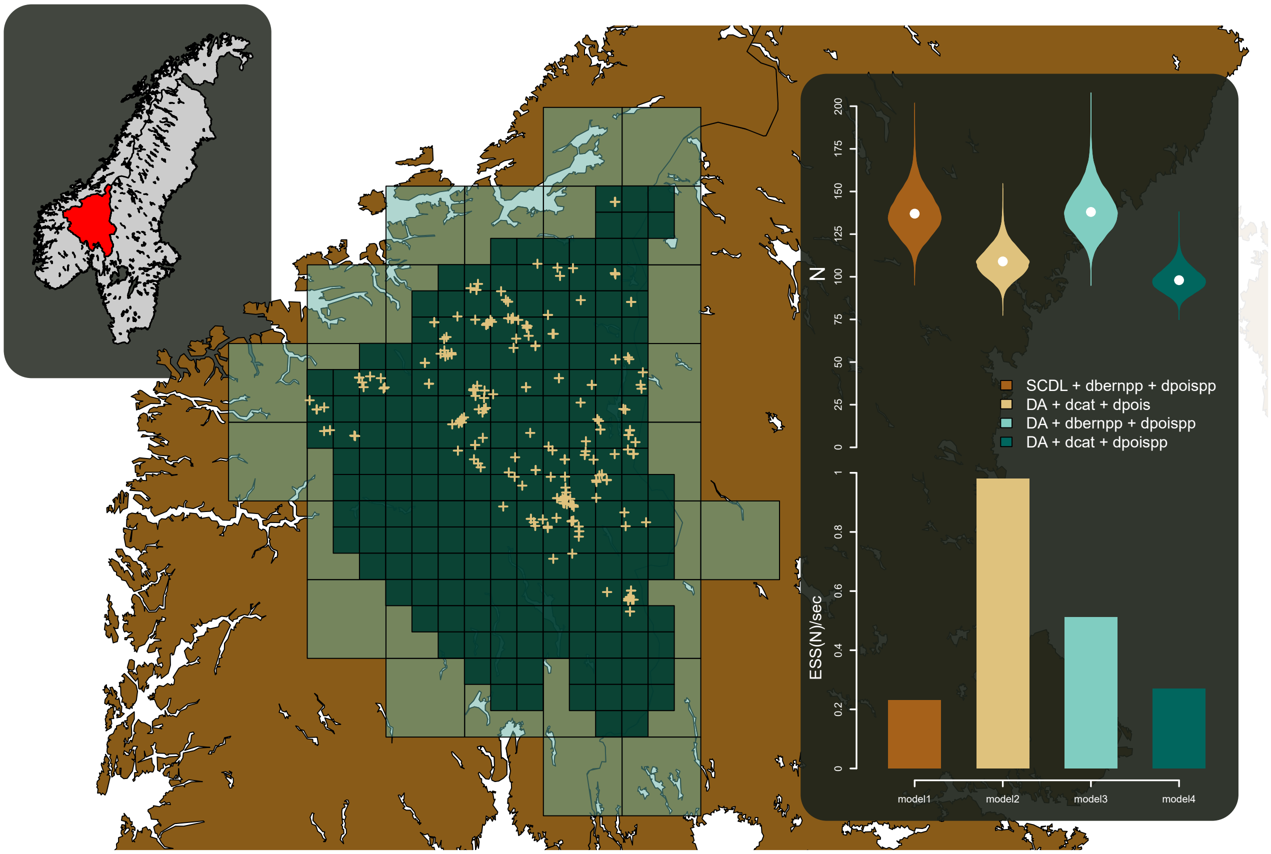

Figure S1: Distribution of non-invasive DNA samples (yellow crosses) of female wolverines collected during winter 2018/19 in Norway. The area searched for samples is represented by the red polygon on the map in the upper left corner. Detection windows (20km×20km each) are represented by the dark grid cells. Habitat windows (60km×60km each) are represented by the larger and lighter grid cells. The violin plots present the posterior samples of the parameter N for the four different methods used to analyze the data. The bar plots present the effective sample size per second (ESS/s) for N for the four methods.

Table S1: Further estimation results of the wolverine data analysis using method 1 (Bernoulli process (dbernpp) for density, Poisson process (dpoispp) for detection, and semi-complete data likelihood (SCDL) for model fitting).

Parameter	Mean	Median	SD	2.5%	97.5%
θ_1	3.45	3.47	0.84	1.75	5.01
θ_2	3.69	3.70	0.21	3.28	4.10
θ_3	3.99	3.99	0.14	3.71	4.26
θ_4	0.26	0.26	0.09	0.08	0.43
θ_5	−0.13	−0.13	0.09	−0.32	0.05
θ_6	0.15	0.14	0.10	−0.06	0.34
β_0	0.97	0.97	0.15	0.66	1.25
β_1	0.64	0.64	0.11	0.41	0.85

Table S2: Further estimation results of the wolverine data analysis using method 2 (categorical distribution (dcat) for density, discrete detector approach (dpois) for detection, and data augmentation (DA) for model fitting).

Parameter	Mean	Median	SD	2.5%	97.5%
θ_1	−2.01	−1.95	0.65	−3.42	−0.92
θ_2	−0.93	−0.93	0.23	−1.38	−0.50
θ_3	−0.82	−0.81	0.12	−1.07	−0.58
θ_4	0.41	0.41	0.06	0.29	0.53
θ_5	−0.07	−0.07	0.09	−0.25	0.10
θ_6	0.03	0.03	0.09	−0.15	0.22
β_1	0.57	0.57	0.12	0.33	0.79
ψ	0.36	0.36	0.04	0.29	0.45

Table S3: Further estimation results of the wolverine data analysis using method 3 (Bernoulli process (dbernpp) for density, Poisson process (dpoispp) for detection, and data augmentation (DA) for model fitting).

Parameter	Mean	Median	SD	2.5%	97.5%
θ_1	3.43	3.44	0.84	1.69	5.04
θ_2	3.68	3.69	0.21	3.24	4.09
θ_3	3.98	3.98	0.14	3.70	4.26
θ_4	0.26	0.26	0.09	0.09	0.43
θ_5	−0.13	−0.13	0.09	−0.32	0.05
θ_6	0.14	0.14	0.10	−0.06	0.34
β_1	0.64	0.64	0.11	0.43	0.86
ψ	0.46	0.46	0.05	0.37	0.57

Table S4: Further estimation results of the wolverine data analysis using method 4 (categorical distribution (dcat) for density, Poisson process (dpoispp) for detection, and data augmentation (DA) for model fitting).

Parameter	Mean	Median	SD	2.5%	97.5%
θ_1	0.43	0.47	0.65	−0.99	1.55
θ_2	1.33	1.34	0.21	0.93	1.73
θ_3	1.47	1.48	0.12	1.24	1.70
θ_4	0.42	0.42	0.06	0.30	0.54
θ_5	0.01	0.02	0.09	−0.16	0.18
θ_6	0.00	0.00	0.09	−0.18	0.18
β_1	15.14	15.51	5.09	3.81	24.78
ψ	0.33	0.33	0.04	0.26	0.40

References

Zhang, W., J. D. Chipperfield, J. B. Illian, P. Dupont, C. Milleret, P. de Valpine, and R. Bischof. 2022. A flexible and efficient Bayesian implementation of point process models for spatial capture-recapture data. Zenodo. https://doi.org/10.5281/zenodo.7038425.
