# Supplementary material for: A flexible and efficient Bayesian implementation of point process models for spatial capture–recapture data
Source: Ecology. 2022 Nov 30;104(1):e3887. doi: 10.1002/ecy.3887 (PMC10078592; doi:10.1002/ecy.3887)
Supplement: Supplementary file 3 — Appendix S3 [file ECY-104-0-s002.pdf]

Appendix S3: A flexible and efficient Bayesian implementation of point process models for spatial capture-recapture data

Zhang W., J.D. Chipperfield, J.B. Illian, P. Dupont, C. Milleret,
P. de Valpine, R. Bischof

Ecology

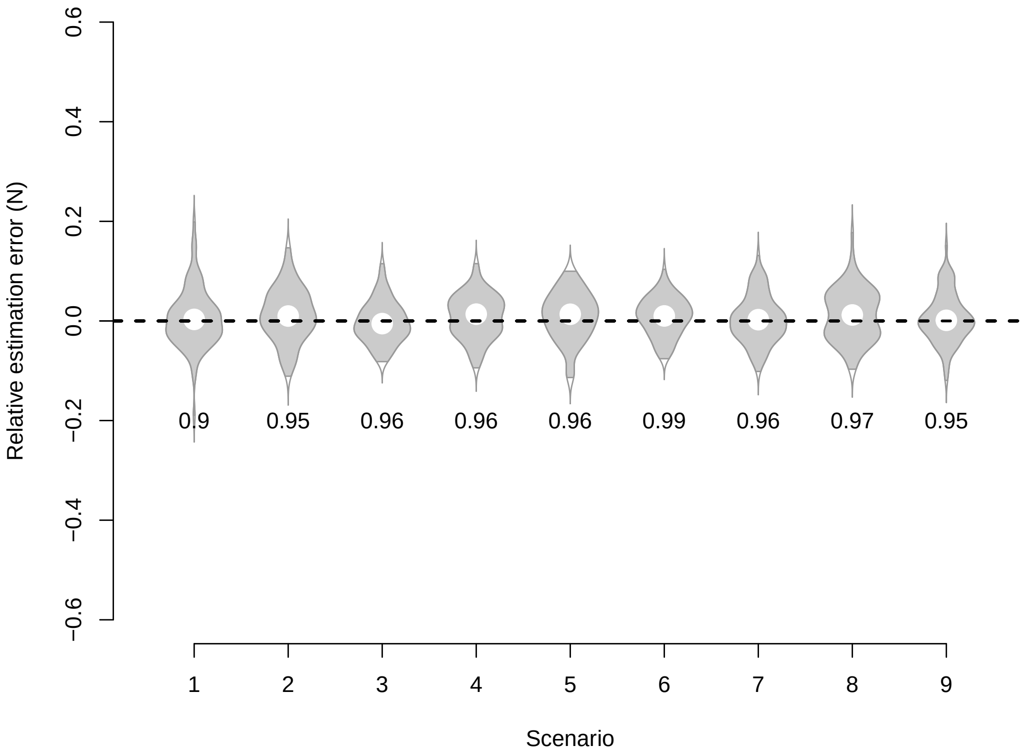

Figure S1: Violin plots showing the distribution of relative estimation error (defined as (estimate − true value) / true value) of the population size N based on 100 replicates for each scenario. The values of (β_1, θ_1) in scenarios 1–9 are $(-1,-1), (0,-1), (1,-1), (-1,0), (0,0), (1,0), (-1,1), (0,1)$, and (1, 1) respectively. The value below each violin plot gives the credible interval coverage.
